# Assessment of ADHD Knowledge, Awareness, and Beliefs Among Parents of Children and Adolescents With ADHD in the Riyadh Region, Saudi Arabia

**DOI:** 10.7759/cureus.115587

**Published:** 2026-09-01

**Authors:** Khaleel Alyahya, Shatha M Alshabani, Lara A Alageel, Razan S Alasmari, Talat E Shaheen, Atheer A AlKanhal, Aljazi Albabtain

**Affiliations:** 1 Department of Anatomy, King Saud University Medical City, Riyadh, SAU; 2 College of Medicine, King Saud University, Riyadh, SAU

**Keywords:** attention-deficit/hyperactivity disorder, attitudes, health knowledge, health literacy, parental awareness, parental beliefs, parental knowledge, parents, practice, saudi arabia

## Abstract

Background

Attention-deficit/hyperactivity disorder (ADHD) is a common neurodevelopmental disorder, and adequate parental knowledge is important for recognizing symptoms and supporting appropriate management. This study assessed parental knowledge, awareness, and beliefs regarding ADHD in Riyadh, Saudi Arabia.

Methods

A cross-sectional study was conducted among parents of children and adolescents with ADHD residing in the Riyadh Region between September 2023 and May 2024. Data were collected anonymously using a 32-item self-administered online questionnaire. Descriptive statistics and chi-square tests were used to assess associations between parental characteristics and knowledge and belief categories.

Results

A total of 388 parents participated, of whom 84.3% were mothers. Most participants demonstrated good recognition of common ADHD symptoms; 95.6% recognized difficulties with maintaining attention, and 92.8% recognized difficulties remaining focused. However, misconceptions remained common, with 68.3% believing that individuals with ADHD are always hyperactive and only 41.5% correctly recognizing that childhood ADHD medication use does not increase the risk of drug abuse during adolescence. More favorable knowledge/awareness was significantly associated with parental sex, age, educational level, and child sex, while more favorable beliefs were associated with parental sex and educational level. Additionally, 94.6% of parents reported a need for additional ADHD awareness campaigns.

Conclusion

Although parents demonstrated good recognition of common ADHD symptoms, important misconceptions regarding ADHD and its management remain. Targeted parent education and awareness programs may help address these knowledge gaps and support better-informed care for children and adolescents with ADHD.

## Introduction

Attention-deficit/hyperactivity disorder (ADHD) is a neurodevelopmental disorder characterized by persistent patterns of inattention, hyperactivity, and impulsivity that are more pronounced than typically expected for children of the same age and can significantly affect daily functioning [1]. ADHD is one of the most common neurobehavioral disorders in children [2], with a global prevalence of 3.4% [3] and a rate of 12.4% in Saudi Arabia [4]. The prevalence of the inattentive and hyperactive subtypes was 2.9% and 2.5%, respectively, with 2.5% experiencing both. No significant sex differences in ADHD rates were found [4].

Research highlights the importance of parents having adequate knowledge and a supportive attitude toward ADHD, enabling better involvement in treatment and appropriate responses to their child’s condition [1,2]. However, many parents still lack sufficient understanding of ADHD [1,3-8] and often hold misconceptions [9]. Studies comparing mothers’ and fathers’ knowledge yielded mixed findings: one showed mothers had greater awareness [8], while the other found no notable difference [3]. About one-third of parents questioned the accuracy of their child’s ADHD diagnosis [1]. While most (81.9%) believed ADHD would not resolve without intervention, nearly one-third saw it as a temporary childhood habit [3]. Parents often associate ADHD with hyperactivity, overlooking the inattentive type [1,6]. One study noted that delays in seeking medical help were often due to mothers not recognizing their child’s symptoms or being unsure where to seek assistance [10].

Studies have shown that many parents lack clarity about treatment options [1,3,4,6,7], leading to hesitation about using medication. Uncertainty about treatment duration and adjustments during adolescence is also common [7].

Several factors, including the parent-child relationship [8,11], parental education, child age, sex, grade, and sources of information, have been associated with parental knowledge and awareness. Parents with greater awareness often obtain information from television, online platforms, healthcare professionals, or personal networks [8]. Higher parental awareness has also been associated with better recognition of ADHD symptoms, while greater parental education and having older children have been linked to higher levels of knowledge [8].

Parental beliefs about the causes of ADHD vary across regions. In the U.S., 25% of parents linked ADHD in adolescents to sugar intake [9]. In South Africa, 92.4% blamed sugar and food additives, while 78.5% viewed ADHD as misbehavior rather than a neurodevelopmental condition [8].

Despite the recognized prevalence and impact of ADHD in Saudi Arabia, limited evidence is available regarding parental knowledge, awareness, and beliefs about ADHD, specifically in the Riyadh Region. Existing studies have examined parental understanding and perceptions of ADHD in other populations and settings, but regional evidence from Riyadh remains limited. In addition, the extent to which parental demographic characteristics are associated with knowledge and beliefs, as well as the sources of ADHD-related information used by parents in this population, has not been sufficiently characterized. Therefore, this study aimed to assess parental knowledge and awareness of ADHD, examine parental beliefs regarding its etiology and management, identify common sources of ADHD-related information, and evaluate the associations between these domains and selected parental and child demographic characteristics among parents of children and adolescents with ADHD in the Riyadh Region, Saudi Arabia.

## Materials and methods

Study design and participants

A cross-sectional study was conducted among parents of children and adolescents with ADHD residing in the Riyadh Region, Saudi Arabia, between September 2023 and May 2024. Both Saudi citizens and non-Saudi residents were eligible to participate.

Parents were included if they resided in the Riyadh Region, had a child or adolescent aged 1-18 years with ADHD, and had access to the online questionnaire. ADHD status was based on parental report of a clinically diagnosed ADHD condition; diagnostic records were not independently verified by the research team. Parents who did not reside in the Riyadh Region or whose child was outside the specified age range were excluded.

Sample size and sampling

The required sample size was calculated using a single-proportion formula, yielding an initial estimate of 384 participants. An additional 20% was added to account for potential non-response, resulting in a target sample size of 461 participants. Participants were recruited using convenience sampling. The questionnaire was distributed electronically through Google Forms and disseminated primarily via Telegram and WhatsApp.

Questionnaire development and data collection

Data were collected using a self-administered, 32-item online questionnaire developed by the research team based on the study objectives and relevant literature. The questionnaire consisted of four sections assessing: (1) sociodemographic characteristics; (2) parental knowledge and awareness of ADHD; (3) sources of ADHD-related information; and (4) parental beliefs regarding the etiology and management of ADHD (Appendices).

The questionnaire was pretested among parents of children and adolescents with ADHD residing in Riyadh to assess the clarity, comprehensibility, and relevance of the questions. Feedback obtained during pretesting was used to refine the questionnaire before the main data collection.

As the questionnaire was developed specifically for this study, it had not undergone formal psychometric validation, and a formal reliability analysis, including calculation of Cronbach’s alpha, was not performed. The questionnaire was pretested among parents of children and adolescents with ADHD residing in Riyadh to assess clarity, comprehensibility, and relevance, and the feedback was used to refine the questionnaire before the main data collection.

For the purposes of this study, knowledge and awareness referred to participants’ understanding and recognition of ADHD-related symptoms, characteristics, and clinical information, as assessed by the relevant questionnaire items. Beliefs referred to participants’ perceptions regarding the etiology, nature, and management of ADHD. These domains were assessed separately using the corresponding questionnaire items.

Scoring and categorization

Knowledge and awareness were assessed based on the number of correct responses to the relevant questionnaire items. The knowledge and awareness score was calculated as the proportion of correctly answered items among the relevant knowledge and awareness items and expressed as a percentage. Participants who obtained ≥50% correct responses were categorized as having more favorable knowledge and awareness, whereas those who obtained <50% correct responses were categorized as having less favorable knowledge and awareness. Parental beliefs regarding ADHD were assessed based on the number of favorable responses to the relevant belief items. The belief score was calculated as the proportion of favorable responses among the relevant belief items and expressed as a percentage. Participants with ≥50% favorable responses were categorized as having more favorable beliefs, whereas those with <50% favorable responses were categorized as having less favorable beliefs.

Ethical considerations

Ethical approval was obtained from the Institutional Review Board of King Saud University. Participation was voluntary, and responses were collected anonymously. No incentives were provided.

Statistical analysis

Data were analyzed using IBM SPSS Statistics version 26.0 (IBM Corp., Armonk, NY, US). Categorical variables were presented as frequencies and percentages, while continuous variables were summarized using means and standard deviations. Independent-samples t-tests were used to compare continuous variables between two groups, while one-way analysis of variance (ANOVA) was used to compare continuous variables across three or more groups, where appropriate. Pearson’s chi-square tests were used to assess associations between categorical variables, and odds ratios were calculated where appropriate. A p-value of <0.05 was considered statistically significant.

## Results

A total of 388 parents of children with ADHD participated in the current study, all of whom were from Riyadh. The majority of participants were mothers (84.3%) and married (87.1%), with the largest proportion (46.1%) aged 30-39 years. Most parents (64.2%) had a college education. Regarding the children’s characteristics, 66.8% were male, and 84.3% were aged 1-12 years. The results are presented in Table 1.

**Table 1:** Demographic characteristics of the study participants

Variable	Category	n	%
Parent sex	Father	61	15.7
Parent sex	Mother	327	84.3
Parent age	20–29 years old	41	10.6
Parent age	30–39 years old	179	46.1
Parent age	40–49 years old	135	34.8
Parent age	50–59 years old	30	7.7
Parent age	60 years and above	3	0.8
Marital status	Married	338	87.1
Marital status	Divorced	42	10.8
Marital status	Widowed	8	2.1
Parent educational level	High school or less	77	19.8
Parent educational level	College level	249	64.2
Parent educational level	Higher educational level	62	16.0
Child/adolescent sex	Male	259	66.8
Child/adolescent sex	Female	129	33.2
Child/adolescent age	Child (1–12 years old)	327	84.3
Child/adolescent age	Adolescent (13–18 years old)	61	15.7

Parents’ awareness of ADHD is presented in Table 2. More than two-thirds (69.8%) of parents reported having sufficient knowledge of ADHD to enable them to interact appropriately with their child or adolescent. However, 68.3% incorrectly believed that individuals with ADHD are always hyperactive, while 65.7% correctly recognized that ADHD is not associated with low IQ scores. Additionally, 67.8% recognized that ADHD can affect both children and adolescents, and 84.3% recognized that individuals with ADHD often experience greater challenges in their relationships with classmates. Regarding the ability to remain seated when asked, 84.8% of participants agreed that individuals with ADHD may have difficulty sitting still. The majority of parents (95.6%) recognized that individuals with ADHD have difficulties maintaining attention, while 92.8% recognized that they are easily distracted and have difficulty remaining focused on a single task. More than half (54.4%) of the participants believed that children with ADHD should not be punished in the same way as other children when they behave inappropriately, and 70.9% correctly recognized that individuals with ADHD can achieve the same level of success as others. Only 41.5% of parents correctly recognized that the use of ADHD medication during childhood does not increase the risk of drug abuse during adolescence.

**Table 2:** Parents’ knowledge and awareness regarding ADHD

Questionnaire item	Response	n	%
As a parent, I have sufficient knowledge about ADHD that enables me to interact with my child in an appropriate manner	Agree	271	69.8
As a parent, I have sufficient knowledge about ADHD that enables me to interact with my child in an appropriate manner	Disagree	68	17.5
As a parent, I have sufficient knowledge about ADHD that enables me to interact with my child in an appropriate manner	I don't know	49	12.6
Patients with ADHD are always hyperactive	Agree	265	68.3
Patients with ADHD are always hyperactive	Disagree	113	29.1
Patients with ADHD are always hyperactive	I don't know	10	2.6
Patients with ADHD have lower IQ scores compared to non-ADHD children	Agree	104	26.8
Patients with ADHD have lower IQ scores compared to non-ADHD children	Disagree	255	65.7
Patients with ADHD have lower IQ scores compared to non-ADHD children	I don't know	29	7.5
Patients with ADHD often face more challenges in their relationships with classmates	Agree	327	84.3
Patients with ADHD often face more challenges in their relationships with classmates	Disagree	47	12.1
Patients with ADHD often face more challenges in their relationships with classmates	I don't know	14	3.6
ADHD is a disorder that affects children only	Agree	59	15.2
ADHD is a disorder that affects children only	Disagree	263	67.8
ADHD is a disorder that affects children only	I don't know	66	17.0
ADHD patients find it difficult to sit still when they are asked to	Agree	329	84.8
ADHD patients find it difficult to sit still when they are asked to	Disagree	47	12.1
ADHD patients find it difficult to sit still when they are asked to	I don't know	12	3.1
ADHD patients have difficulty staying focused on one task, and they frequently shift between different tasks	Agree	360	92.8
ADHD patients have difficulty staying focused on one task, and they frequently shift between different tasks	Disagree	14	3.6
ADHD patients have difficulty staying focused on one task, and they frequently shift between different tasks	I don't know	14	3.6
ADHD patients have difficulties with maintaining attention and are easily distracted	Agree	371	95.6
ADHD patients have difficulties with maintaining attention and are easily distracted	Disagree	12	3.1
ADHD patients have difficulties with maintaining attention and are easily distracted	I don't know	5	1.3
ADHD patients often interrupt others	Agree	332	85.6
ADHD patients often interrupt others	Disagree	36	9.3
ADHD patients often interrupt others	I don't know	20	5.2
ADHD patients have learning difficulties in comparison to their peers	Agree	305	78.6
ADHD patients have learning difficulties in comparison to their peers	Disagree	57	14.7
ADHD patients have learning difficulties in comparison to their peers	I don't know	26	6.7
ADHD patients are not subject to punishment as other kids when they act inappropriately	Agree	211	54.4
ADHD patients are not subject to punishment as other kids when they act inappropriately	Disagree	128	33.0
ADHD patients are not subject to punishment as other kids when they act inappropriately	I don't know	49	12.6
ADHD patients can achieve the same level of success in life as others	Agree	275	70.9
ADHD patients can achieve the same level of success in life as others	Disagree	59	15.2
ADHD patients can achieve the same level of success in life as others	I don't know	54	13.9
ADHD medication use in childhood increases the risk of drug abuse in adolescence	Agree	54	13.9
ADHD medication use in childhood increases the risk of drug abuse in adolescence	Disagree	161	41.5
ADHD medication use in childhood increases the risk of drug abuse in adolescence	I don't know	173	44.6

Comparisons of knowledge/awareness levels regarding ADHD according to the demographic characteristics of the study participants are presented in Table 3. Mothers had a significantly greater proportion in the more favorable knowledge/awareness category than fathers (50.2% vs. 26.2%, p = 0.001). Participants aged 30-39 years had the highest proportion in this category compared with the other age groups (p = 0.023). Educational attainment was also significantly associated with knowledge/awareness level, with 59.7% of participants with higher education classified in the more favorable category compared with 35.1% of those with a high school education or less (p = 0.015). Parents of male children with ADHD also had a significantly higher proportion in the more favorable knowledge/awareness category than parents of female children (53.7% vs. 31.8%, p < 0.0001).

**Table 3:** Comparison of knowledge levels regarding ADHD according to demographic characteristics Pearson’s chi-square test was used.

Variable	Category	More favorable responses (n)	Less favorable responses (n)	Statistical test
Parent sex	Father	16	45	Pearson χ² = 11.831; p = 0.0006
Parent sex	Mother	164	163	-
Parent age	20–29 years old	13	28	Pearson χ² = 9.525; p = 0.023
Parent age	30–39 years old	97	82	-
Parent age	40–49 years old	57	78	-
Parent age	50 years and above	13	20	-
Marital status	Married	159	179	Pearson χ² = 0.523; p = 0.770
Marital status	Divorced	18	24	-
Marital status	Widowed	3	5	-
Parent educational level	High school or less	27	50	Pearson χ² = 8.376; p = 0.015
Parent educational level	College level	116	133	-
Parent educational level	Higher educational level	37	25	-
Child/adolescent sex	Male	139	120	Pearson χ² = 16.584; p < 0.0001
Child/adolescent sex	Female	41	88	-
Child/adolescent age	Child (1–12 years old)	152	175	Pearson χ² = 0.007; p = 0.933
Child/adolescent age	Adolescent (13–18 years old)	28	33	-

Regarding parental beliefs about ADHD, 51.3% of participants believed that ADHD is a genetic disorder, while 40.7% believed that children who consume large amounts of candy and sugar are at increased risk of developing ADHD. More than half (55.7%) did not believe that the way a child is raised could cause ADHD, while 69.6% correctly identified ADHD as a neurodevelopmental disorder rather than a behavioral issue. Furthermore, 70.4% recognized that ADHD is a lifelong condition, although its symptoms can be managed. Most parents (81.2%) recognized a combination of behavioral therapy and medication as the most effective treatment approach. The results are presented in Table 4.

**Table 4:** Parents’ beliefs regarding the etiology and management of ADHD

Variable	Response	n	%
Believing that ADHD is a genetic disorder	Yes	199	51.3
Believing that ADHD is a genetic disorder	No	85	21.9
Believing that ADHD is a genetic disorder	I don't know	104	26.8
Thinking that children who eat lots of candy and sugar are at risk of having ADHD	Yes	158	40.7
Thinking that children who eat lots of candy and sugar are at risk of having ADHD	No	164	42.3
Thinking that children who eat lots of candy and sugar are at risk of having ADHD	I don't know	66	17.0
Thinking that the way the child is raised can be a cause of ADHD	Yes	109	28.1
Thinking that the way the child is raised can be a cause of ADHD	No	216	55.7
Thinking that the way the child is raised can be a cause of ADHD	I don't know	63	16.2
Thinking that ADHD is a behavioral problem or a neurodevelopmental disorder	It is a misbehaviour disorder	46	11.9
Thinking that ADHD is a behavioral problem or a neurodevelopmental disorder	It is a neurodevelopmental disorder	270	69.6
Thinking that ADHD is a behavioral problem or a neurodevelopmental disorder.	I don't know	72	18.6
Understanding of the course of ADHD	ADHD can be cured completely	51	13.1
Understanding of the course of ADHD	ADHD is a life-long disorder	24	6.2
Understanding of the course of ADHD	It is a permanent disorder but the symptoms can be managed	273	70.4
Understanding of the course of ADHD	I don't know	40	10.3
Their knowledge about what is more effective regarding the treatment	Only behavioural therapy is effective	26	6.7
Their knowledge about what is more effective regarding the treatment.	Only medications are effective	7	1.8
Their knowledge about what is more effective regarding the treatment.	Both behavioural therapy and medications are effective depending on the patient’s condition	315	81.2
Their knowledge about what is more effective regarding the treatment.	I don't think that behavioural therapy nor medications are effective	17	4.4
Their knowledge about what is more effective regarding the treatment.	I don't know	23	5.9
Thinking that children with ADHD have comorbid mental health conditions, e.g., anxiety, depression, etc.	Yes	262	67.5
Thinking that children with ADHD have comorbid mental health conditions, e.g., anxiety, depression, etc.	No	42	10.8
Thinking that children with ADHD have comorbid mental health conditions, e.g., anxiety, depression, etc.	I don't know	84	21.6

The association between parental beliefs regarding ADHD and participant characteristics is presented in Table 5. The less favorable belief category was more frequently observed among fathers (80.3%) and participants with a high school education or less (80.5%), with statistically significant associations (p = 0.02 and p = 0.001, respectively). No statistically significant associations were observed between parental beliefs and the other demographic characteristics examined.

**Table 5:** Comparison of parental beliefs regarding ADHD according to demographic characteristics Pearson’s chi-square test was used.

Variable	Category	More favorable (n)	Less favorable (n)	Statistical test
Parent sex	Father	12	49	Pearson χ² = 5.410; p = 0.020
Parent sex	Mother	114	213	-
Parent age	20–29 years old	15	26	Pearson χ² = 4.378; p = 0.223
Parent age	30–39 years old	66	113	-
Parent age	40–49 years old	36	99	-
Parent age	50 years and above	9	24	-
Marital status	Married	116	222	Pearson χ² = 5.810; p = 0.055
Marital status	Divorced	10	32	-
Marital status	Widowed	0	8	-
Parent educational level	High school or less	15	62	Pearson χ² = 13.088; p = 0.001
Parent educational level	College level	81	168	-
Parent educational level	Higher educational level	30	32	-
Child/adolescent sex	Male	91	168	Pearson χ² = 2.515; p = 0.113
Child/adolescent sex	Female	35	94	-
Child/adolescent age	Child (1–12 years old)	112	215	Pearson χ² = 2.994; p = 0.084
Child/adolescent age	Adolescent (13–18 years old)	14	47	-

When knowledge/awareness and parental beliefs regarding ADHD were considered together, mothers had a significantly greater proportion in the more favorable category than fathers (47.1% vs. 19.7%, p < 0.0001). Participants aged 30-39 years had the highest proportion in the more favorable category, with a statistically significant association (p = 0.027). Educational attainment was also significantly associated with the combined knowledge/awareness and belief category, with 58.1% of highly educated parents classified in the more favorable category compared with 29.9% of those with a high school education or lower (p = 0.004). Additionally, parents of male children had a significantly higher proportion in the more favorable category than parents of female children (49.0% vs. 30.2%, p < 0.0001). The data are presented in Table 6.

**Table 6:** Comparison of knowledge and beliefs regarding ADHD according to demographic characteristics Pearson’s chi-square test was used.

Variable	Category	More favorable knowledge and beliefs (n)	Less favorable knowledge and beliefs (n)	Statistical test
Parent sex	Father	12	49	Pearson χ² = 15.793; p < 0.0001
Parent sex	Mother	154	173	-
Parent age	20–29 years old	17	24	Pearson χ² = 9.202; p = 0.027
Parent age	30–39 years old	90	89	-
Parent age	40–49 years old	50	85	-
Parent age	50 years and above	9	24	-
Marital status	Married	151	187	Pearson χ² = 5.023; p = 0.081
Marital status	Divorced	14	28	-
Marital status	Widowed	1	7	-
Parent educational level	High school or less	23	54	Pearson χ² = 11.163; p = 0.004
Parent educational level	College level	107	142	-
Parent educational level	Higher educational level	36	26	-
Child/adolescent sex	Male	127	132	Pearson χ² = 12.436; p = 0.0004
Child/adolescent sex	Female	39	90	-
Child/adolescent age	Child (1–12 years old)	147	180	Pearson χ² = 4.003; p = 0.045
Child/adolescent age	Adolescent (13–18 years old)	19	42	-

Regarding sources of information about ADHD, more than half (51.0%) of the participants reported that the resources they used did not fully address their questions about ADHD, while 56.4% reported that sufficient and accessible resources covering ADHD were not available. The vast majority (94.6%) of participants believed that there was a need for additional campaigns to raise awareness about ADHD, and 91% reported that their level of knowledge and awareness about ADHD had increased after their child was diagnosed with ADHD. The data are presented in Table 7.

**Table 7:** Sources of information and perceived adequacy of ADHD-related resources

Variable	Response	n	%
Do the resources that you use fulfil all your queries about ADHD?	Yes	152	39.2
Do the resources that you use fulfil all your queries about ADHD?	No	198	51.0
Do the resources that you use fulfil all your queries about ADHD?	I don't know	38	9.8
Do you believe that there are sufficient accessible resources that cover the topic of ADHD?	Yes	117	30.2
Do you believe that there are sufficient accessible resources that cover the topic of ADHD?	No	219	56.4
Do you believe that there are sufficient accessible resources that cover the topic of ADHD?	I don't know	52	13.4
Do you believe that there is a need for more campaigns that raise awareness about ADHD?	Yes	367	94.6
Do you believe that there is a need for more campaigns that raise awareness about ADHD?	No	12	3.1
Do you believe that there is a need for more campaigns that raise awareness about ADHD?	I don't know	9	2.3
Do you think that your level of knowledge and awareness about ADHD have increased after your child was diagnosed with ADHD?	Yes	353	91.0
Do you think that your level of knowledge and awareness about ADHD have increased after your child was diagnosed with ADHD?	No	22	5.7
Do you think that your level of knowledge and awareness about ADHD have increased after your child was diagnosed with ADHD?	I don't know	13	3.4

## Discussion

We conducted this study to assess the awareness and knowledge of parents of children and adolescents diagnosed with ADHD in the Riyadh Region of Saudi Arabia. The findings showed that nearly one-third of parents did not report having sufficient knowledge about ADHD. This is consistent with a similar study conducted in Tehran, which found that many parents were poorly informed about ADHD [1]. These findings suggest that gaps in parental understanding of ADHD remain present across different populations and settings.

Previous research [1,2] reported that 62% of parents did not associate ADHD with high IQ. In our study, 65.7% of parents correctly recognized that ADHD is not associated with low IQ scores. Additionally, a prior study concluded that ADHD manifests similarly across children with high, normal, and low IQ, although having a high IQ may positively influence certain outcomes such as reading achievement [3]. These findings indicate that parents in our study demonstrated relatively good recognition of the relationship between ADHD and intellectual ability.

Our results revealed that children with ADHD frequently encountered greater challenges in their relationships with their classmates. In the current study, 84.3% of parents recognized that children with ADHD may experience greater challenges in their relationships with classmates. Gresham et al. [4] found that 70% of elementary school children with comorbid ADHD and conduct problems had no reciprocal friendships in their classroom. Additionally, several studies suggest that these reciprocal friendships tend to be very short-lived [5]. The similarity between these findings highlights that social difficulties may represent an important aspect of ADHD that parents recognize in addition to its more commonly recognized behavioral symptoms.

Most parents demonstrated good recognition of common ADHD symptoms. In particular, 84.8% recognized that individuals with ADHD may have difficulty sitting still, while 95.6% and 92.8% recognized difficulties with maintaining attention and remaining focused, respectively. These findings suggest that parents were relatively familiar with the core behavioral and attentional manifestations of the disorder, consistent with the established clinical features of ADHD [6,10-12]. The relatively high recognition of these symptoms may reflect their more observable nature in children’s daily lives.

However, an important knowledge gap was observed regarding ADHD treatment and subsequent substance use. Stimulant medication used to treat young children with ADHD does not affect the risk of substance abuse in adulthood, either increasing or decreasing it [13]. Other research suggests that ADHD pharmacotherapy, typically with stimulants, does not raise the risk of substance abuse, but may actually reduce it [14]. However, in our study, only 41.5% of parents correctly recognized that ADHD medication use during childhood does not increase the risk of drug abuse during adolescence, suggesting a considerable gap in parental knowledge regarding the relationship between ADHD treatment and subsequent substance use. This difference between parental perceptions and findings from previous research highlights an area in which additional information may be beneficial.

Consistent with the findings of the current study, previous research has shown that higher parental educational attainment is associated with greater awareness and knowledge of ADHD [1,15]. In our study, parents with higher education were more likely to fall into the more favorable knowledge/awareness category than those with a high school education or less. The consistency between our findings and previous research suggests that parental educational attainment may be an important factor associated with ADHD-related knowledge and awareness.

Parental beliefs regarding the causes of ADHD also showed differences compared with previous findings. In a 2007 study conducted in Shiraz [1], 52% of parents attributed ADHD to poor parenting, while 47.7% believed that it was due to genetic or biological vulnerabilities. In contrast, in our study, 55.7% of parents did not believe that the way a child is raised could cause ADHD, while 51.3% believed that ADHD has a genetic basis. These findings highlight that misconceptions regarding the causes of ADHD remain present among a proportion of parents. The differences between the findings may reflect differences in the populations studied or the settings in which the studies were conducted.

Previous studies have identified several potential causes of ADHD, including food additives [16], environmental factors [17], and sugar consumption [18]. However, Del-Ponte et al. found no link between sucrose intake between ages 6 and 11 and the development of ADHD [19]. There is conflicting evidence in the literature regarding the effect of sugar on ADHD occurrence [19]. In our study, 40.7% of the participants believed that children who consume large amounts of candy and sugar are at a higher risk of developing ADHD. This finding suggests that beliefs regarding dietary factors remain present among a proportion of parents despite the available evidence, highlighting another area of potential knowledge gaps.

Limitations

This study has several limitations. First, it was conducted among parents of children with ADHD in the Riyadh Region, which may limit the generalizability of the findings to other regions of Saudi Arabia. Second, the study questionnaire was developed specifically for this study based on the literature and pretested to assess clarity, comprehensibility, and relevance; however, it had not undergone formal psychometric validation or reliability testing, including calculation of Cronbach’s alpha. This may limit the robustness of the measurement of parental knowledge, awareness, and beliefs. Third, the cross-sectional design provides a snapshot of parental knowledge and beliefs and identifies associations but cannot establish causal relationships. Future studies should include larger, more geographically diverse samples and use formally validated and reliable instruments to improve the reliability and generalizability of the findings.

## Conclusions

This study found that parents of children with ADHD in the Riyadh Region demonstrated awareness of several common features of ADHD, but gaps and misconceptions regarding its causes, treatment, and medication remained. Knowledge and awareness were associated with parental sex, age, educational attainment, and child sex. The findings also highlight a substantial need for accessible and reliable ADHD information. Targeted parent education and awareness initiatives may help address these gaps and provide families with evidence-based information to better support children with ADHD.

ADHD-specific educational programs and accessible, evidence-based resources are recommended to address parental knowledge gaps and misconceptions regarding its causes, symptoms, treatment, and medication. These initiatives can be delivered through healthcare and community settings. Future research should include larger, geographically diverse samples across Saudi Arabia and use validated assessment tools to evaluate parental knowledge and beliefs and identify regional differences.

## Appendices

Questionnaire 

**Table 8:** Sociodemographic information

No.	Question / item	Response options
1	Are you a parent of a child with ADHD in Riyadh Region?	Yes / No
2	The gender of the parent:	A Father / A Mother
3	Age of the parent:	20-29 years old / 30-39 years old / 40-49 years old / 50-59 years old / 60 years and above
4	The marital status:	Married / Divorced / Widowed
5	The educational level of the parent:	High school or less / College level / Higher educational level
6	The gender of the child/adolescent:	Male / Female
7	The age of the child/adolescent:	A child (1-12 years old) / An adolescent (13-18 years old)

**Table 9:** Questions that assess the level of awareness among parents

No.	Question / item	Response options
1	As a parent, I have sufficient knowledge about ADHD that enables me to interact with my child in an appropriate manner.	Agree / Disagree / I don’t know
2	Patients with ADHD are always hyperactive.	Agree / Disagree / I don’t know
3	Patients with ADHD have lower IQ scores compared to non-ADHD children.	Agree / Disagree / I don’t know
4	Patients with ADHD often face more challenges in their relationships with classmates.	Agree / Disagree / I don’t know
5	ADHD is a disorder that affects children only.	Agree / Disagree / I don’t know
6	ADHD patients find it difficult to sit still when they are asked to.	Agree / Disagree / I don’t know
7	ADHD patients have difficulty staying focused on one task, and they frequently shift between different tasks.	Agree / Disagree / I don’t know
8	ADHD patients have difficulties with maintaining attention and are easily distracted.	Agree / Disagree / I don’t know
9	ADHD patients often interrupt others.	Agree / Disagree / I don’t know
10	ADHD patients have learning difficulties in comparison to their peers.	Agree / Disagree / I don’t know
11	ADHD patients are not subject to punishment as other kids when they act inappropriately.	Agree / Disagree / I don’t know
12	ADHD patients can achieve the same level of success in life as others.	Agree / Disagree / I don’t know
13	ADHD medication use in childhood increases the risk of drug abuse in adolescence.	Agree / Disagree / I don’t know

**Table 10:** Questions that identify the knowledge sources utilized by the parents

No.	Question / item	Response options
1	What are the resources that you obtain your knowledge about ADHD from?	Internet / Social Media / Books / Television programs / Scientific Magazines / Medical Specialists / Community Members: (family, friends, or co-workers)
2	Do the resources that you use fulfil all your queries about ADHD?	Yes / No / I don’t know
3	Do you believe that there are sufficient accessible resources that cover the topic of ADHD?	Yes / No / I don’t know
4	Do you believe that there is a need for more campaigns that raise awareness about ADHD?	Yes / No / I don’t know
5	Do you think that your level of knowledge and awareness about ADHD have increased after your child was diagnosed with ADHD?	Yes / No / It has not changed / I don’t know

**Table 11:** Questions that assess the beliefs of parents related to the etiology of ADHD

No.	Question / item	Response options
1	Do you believe that ADHD is a genetic disorder?	Yes / No / I don’t know
2	Do you think that children who eat lots of candy and sugar are at risk of having ADHD?	Yes / No / I don’t know
3	Do you think that the way the child is raised can be a cause of ADHD?	Yes / No / I don’t know
4	Do you think that ADHD is a misbehaviour or a neurodevelopmental disorder?	It is a misbehaviour disorder / It is a neurodevelopmental disorder / I don’t know
5	Do you think that ADHD is curable or is a life-long disorder?	ADHD can be cured completely / ADHD is a life-long disorder / It is a permanent disorder but the symptoms can be managed / I don’t know
6	What do you think is more effective regarding the treatment?	Only behavioural therapy is effective / Only medications are effective / Both behavioural therapy and medications are effective depending on the patient’s condition / I don’t think that behavioural therapy nor medications are effective / I don’t know
7	Do you think that children with ADHD have comorbid mental health conditions, e.g., anxiety, depression, etc.	Yes / No / I don’t know
